# The Association Between Persistence and Change in Behavioral Difficulties During Early to Middle Childhood and Cognitive Abilities at Age 8

**DOI:** 10.1007/s10578-022-01453-1

**Published:** 2022-11-14

**Authors:** Denise Neumann, Elizabeth R. Peterson, Lisa Underwood, Susan M.B. Morton, Karen E. Waldie

**Affiliations:** 1 School of Psychology, Faculty of Science, University of Auckland, Private Bag 92019, 1142 Auckland, New Zealand; 2 Centre for Longitudinal Research - He Ara ki Mua, The University of Auckland, Auckland, New Zealand; 3 School of Population Health, The University of Auckland, Auckland, New Zealand

**Keywords:** Growing Up in New Zealand, cohort, longitudinal, cognition, behavior

## Abstract

We investigated the association between persistence and change in behavioral difficulties during early to middle childhood and several cognitive outcomes. We observed 3904 8-year-olds enrolled in the longitudinal study *Growing Up in New Zealand* (50% male/female; 23% Māori, 9% Pacific Peoples, 13% Asian, 2% Middle Eastern/Latin American/African, 9% Other, 43% European). The NIH Toolbox Cognition Battery was used to assess cognitive functioning at 8 years and the Strengths and Difficulties Questionnaire for behavioral difficulties at 4.5 and 8 years. Multivariate logistic regression analyses were conducted, controlling for well-known sociodemographic confounders. Children with persistent or later onset of behavioral difficulties were at higher risk for poorer vocabulary, reading, inhibitory control/attention, episodic memory, working memory and processing speed at age 8 compared to children with no or improved difficulties. Our study supports the importance of addressing both cognitive and behavioral aspects when planning educational programmes and interventions in early and middle childhood.

Abbreviations:

National Institute of Health (NIH)

Cognitive Battery (CB)

Strengths and Difficulties Questionnaire (SDQ)

Data Collection Wave (DCW), Aotearoa New Zealand (NZ).

## Introduction

In recent decades, a considerable amount of research has emerged on the association of behavioral difficulties and the negative impact on a child’s ability to fulfil their educational and developmental potential [1]. Evidence exists that there is a generally a strong link between behavioral problems and cognitive performance in early and middle childhood, [2, 3], notwithstanding that the relationship is likely to be bidirectional in nature [4]. Gremillion and Martel [5] examined associations between language variation and disruptive behavior disorders (DBD) in preschool children. The authors found that children with DBD showed poorer receptive, expressive, and pragmatic language skills compared to children without DBD. Furthermore, preschoolers with increased parent-rated hyperactivity-impulsivity scores demonstrated poorer language skills [5]. Likewise, Sim et al. [6] found language difficulties in preschool children who scored in the abnormal range of the Strengths and Difficulties Questionnaire (SDQ) total difficulties score. A further study compared 7-11-year-olds, who were identified as showing concerning behavior at school, to age- and sex-matched controls. The authors found that children with behavioral concerns were more likely to show structural language, word decoding and pragmatic language difficulties compared with the control group [7].

The link between behavioral difficulties and cognition extends to executive functioning in early and middle childhood. For instance, Raaijmakers and colleagues [72] observed that preschool children with aggressive behavior as indicated by scores at or above the 93rd percentile on the Aggressive Behavior Scale [8] showed inhibition deficits based on six neuropsychological tests [9]. Moreover, Jahromi and Stifter [10] found that preschool children with lower executive functioning on tasks involving inhibition of a prepotent response showed poorer emotion regulation and were less able to control their impulsive behaviors. A further study compared children aged 8–11 years who were identified as hyperactive according to teacher-ratings on the SDQ to matched controls on a battery of cognitive tasks. The authors found that children with hyperactivity performed more poorly on tasks of inhibition and executive function, as well as literacy measures, compared to the control group [11]. Furthermore, hyperactivity and conduct problems measured by the SDQ, have been found to be negatively correlated to reading and arithmetic performance assessed with standardized tests in children aged 3–6 years [12].

A range of longitudinal research has investigated the link between children’s behavior and cognitive functions. For instance, in the *Christchurch Health and Development Study*, Fergusson, Horwood and Lynsky [13] showed that early behavioral tendencies are associated with later developmental outcomes, i.e. conduct problems at age 7–9 years increased the risk of later juvenile offending while attention deficits were related to academic under-achievement in middle childhood. Furthermore, Helland and colleagues [4] looked at language ability in a group of adolescents (12–15 years) with externalising behavior problems compared to a typically developing comparison group. The group with behavioral problems showed poorer language ability and 70% showed language impairments in the clinical range. The authors further found that language and emotional and peer problems assessed with the SDQ in childhood (age 7–9 years), were strongly correlated with language impairments in adolescence (age 12–15 years). The authors concluded that the assessment of language, especially pragmatics, is crucial for follow-up and treatment of behavioral problems in children and adolescents (Helland, Lundervold, Heimann, & Posserud, [4]). McGee et al. [14] investigated to what extent hyperactivity during both the preschool years and at age of school entry leads to later academic and behavioral problems in adolescence by using data from two longitudinal studies, the *Australian Temperament Project* and the *Dunedin Multidisciplinary Health and Development Study*. They found a strong linear relationship between early hyperactivity and continuing school difficulties, attention problems as well as poor reading at age 15.

Existing longitudinal research has mainly focused on how early cognitive difficulties relate to later behavioral problems, rather than vice versa, although the association between cognitive difficulties and behavioral problems is likely to be bidirectional in nature [4, 15, 16]. While some research indicates that behavior problems predispose the child to under-achievement, there is also evidence that language impairments can increase the occurrence of behavioral difficulties [17–19].

In a recent longitudinal study in Aotearoa New Zealand, using data from the large birth cohort study *Growing Up in New Zealand*, D’Souza and colleagues [20] investigated whether persistence and change in serious behavioral problems from ages 2 to 4.5 years is linked to cognitive delay at preschool age in the areas of receptive language, early literacy and executive control. The total difficulties score of the SDQ was used at each time point and children were then categorised as showing no difficulties, improved behavior, concurrent difficulties, and persistent difficulties. The findings indicated that children with concurrent and persistent behavioral difficulties were more likely to show cognitive delays compared to children with no difficulties, after controlling for a range of sociodemographic covariates. They were also at greater risk of having comorbid delays across several cognitive domains. The authors noted that one limitation of the study was that some of the cognitive measures used at 4.5 years were more of an indicator of early cognitive ability and the lack of using a comprehensive neurocognitive battery. Furthermore, it would be of interest to investigate how persistence and change in behavioral difficulties from preschool to school age might have shaped the relationship between behavioral difficulties and cognitive abilities [20].

Transitioning from preschool to school age is an important period for cognitive development. Not only do developmental spurts of key executive functioning take place during this time [21], the transition is also often accompanied by major environmental changes in the children’s lives that may generate new cognitive demands, alongside potential socio-emotional and behavioral challenges [22]. Previous literature shows that while most behavioral difficulties in preschool children tend to be related to developmental phases of testing out boundaries and are no longer evident after transition to school, for some children behavioral difficulties, especially emotional problems, persist during early childhood as well as after school entry, [23–26]. To what extend behavioral difficulties change from early to middle childhood, especially when transitioning into school and the impact this has on cognitive abilities requires further investigation.

To address this and to extend the existing literature, the current study aimed to explore the association between persistence and change in behavioral difficulties administered with the SDQ (when the *Growing Up in New Zealand* children were 4.5 and 8 years of age) and cognitive performance in the comprehensive NIH Toolbox Cognition Battery at 8 years. The cognitive outcomes of interest were comprised of language (reading and vocabulary), executive functioning (inhibitory control/attention and cognitive flexibility) episodic memory, working memory and processing speed.

## Methods

### Participants

Participants were members of the *Growing Up in New Zealand* study, a prospective cohort study with 6822 pregnant women recruited via three contiguous District Health Board regions in Aotearoa New Zealand, who had expected delivery dates between 25th April 2009 and 25th March 2010 [27]. The study’s cohort is broadly generalizable to current births statistics in Aotearoa New Zealand with respect to expected ethnicity, maternal age and parental socioeconomic status at birth [28]. A detailed description of the study’s design and recruitment can be found elsewhere (Morton et al., 2013, 2014). In brief, major data collection waves (DCWs) have included conducting computer assisted telephone and personal interviews to gather information longitudinally relating to six inter-connected domains of child development: health and wellbeing; cognitive and psychosocial; education; family and whānau (extended family); culture and identity; and neighbourhoods and societal context. Participants were included in the current study if complete information was obtained for the cognitive observations and child behavior data at the 4.5 and 8 year DCW (n = 3904).

### Measures

*Cognitive measures.* To assess child cognitive functioning at 8 years, the NIH Toolbox® for Assessment of Neurological and Behavioral Function Cognition Battery (NIH Toolbox CB) was administered [29]. This instrument has been validated against existing gold-standard measures; normed in both English and Spanish languages with a sample of 4,859 participants (age ranges 3–85) (Weintraub et al., 30) and has been validated within the Aotearoa New Zealand context [31]. The NIH Toolbox CB version 7–17 years was delivered to our cohort using the NIH Toolbox iPad app. It comprised seven subtests: Picture Vocabulary Test; Flanker Inhibitory Control and Attention Test; Pattern Comparison Processing Speed Test; List Sorting Working Memory Test; Dimensional Change Card Sort Test; Picture Sequence Memory Test; Oral Reading Recognition Test. Raw scores were used to measure task performance in the present study. Outcomes were dichotomized into children who scored one standard deviation below the mean and all other children.

*Measures of Behavioral Difficulties.* The mother-reported Strengths and Difficulties Questionnaire (SDQ) [32] was used to measure behavioral difficulties when the cohort children were 4.5 and 8 years of age. This questionnaire measures four difficulties subscales (emotional symptoms, peer problems, hyperactivity-inattention and conduct problems) as well as a strengths-based prosocial behavior subscale. The standard parent-report SDQ was used at 4.5 and 8 years. Previous research with the *Growing Up in New Zealand* cohort has shown that the SDQ has good psychometric properties in preschool children [26, 33, 34].

In the current study, the total difficulties score was used, based on the emotional symptoms, peer problems, hyperactivity-inattention and conduct problems subscales with their 5 corresponding items accordingly. The total difficulties score (ranging from 0 to 40) was then converted into categorical variables by using the recommended four band categorisation based on population data, to divide the data into those who are close to average, slightly raised/slightly lowered scores, high/low and very high/very low scores [35]. It is important to note that the reference population data used to determine the categorisation is not based on an Aotearoa New Zealand population. However, the cut-offs used align with those used by the Ministry of Health when reporting on the New Zealand Health Survey [36]. In the current study, the SDQ total difficulties score was further dichotomized into ‘close to average and slightly raised difficulties’ and ‘high and very high’ difficulties [20, 32, 33, 37]. The choice to combine the close to average and slightly raised bands in the current study was due to our interest in the clinically significant high or ‘abnormal’ range, respectively, as per the original 3 band categorization. The high cut-off is typically used to screen for children with significant social and emotional challenges in the nationwide preschool health and development check in Aotearoa New Zealand, known as the B4 School Check [38].

Based on the dichotomized SDQ total difficulties score at 4.5 and 8 years, we derived a persistence/change variable with 4 possible categories: no difficulties (at either time point); improved (behavioral difficulties at 4.5 years but not at 8 years); later onset (no behavioral difficulties at 4.5 years but at 8 years) and persistent (behavioral difficulties at both time points).

*Sociodemographic Covariates.* We controlled for a range of variables that have been found to be related to both behavior and cognitive functioning in children [26, 39–41]. Control variables specific to the child included birthweight, sex, and age in months when assessed at the 8-year DCW. Maternal variables that we controlled for were collected by self-report at the antenatal DCW: mother’s education (no secondary school, secondary school/diploma/trade certificate, Bachelor’s degree or higher); age (< 20 years, 20–29 years; ≥ 30 years); whether or not the pregnancy was planned. At the 4.5 year DCW, mothers reported on their children’s ethnicity by referring to a list of 32 possible answers as well as an open ended ‘Other, please specify’ category (multiple responses were collected). In the current study, Level 1 ethnicity categorisation was utilised, categorised into six categories by external prioritisation according to the Statistics New Zealand priorisation guidelines: Māori; Pacific Peoples; Asian; Middle Eastern, Latin American and African (MELAA); Other; European [42, 43]. We acknowledge that ethnicity is a complex multi-faceted construct which can be understood in different ways and may vary over time which takes careful considerations to capture in a longitudinal study [44–46]. Further, area-level deprivation and rurality at 8 years was also controlled for in the analyses as these have been found to be linked to cognitive functioning in children [47, 48]. To determine SES, the NZDep2013 Index was used, which is an area-level measure using socioeconomic indicators from the 2013 NZ census [49]. Deprivation scores range from least deprived (decile 1) to most deprived (decile 10). In the current study, SES was categorised into high (deciles 8–10), medium (deciles 4–7) and low (deciles 1–3) deprivation. Rurality was defined using Statistics New Zealand’s classification for urban and rural areas [50]. We further controlled for time of testing (finish hour) of the cognitive assessment which might have varied across participants as it was part of a longer face-to-face interview procedure.

### Data Analysis

Analyses were carried out using RStudio version 3.6.1 and IBM SPSS Statistics version 25.0. Statistical significance was given at an *α* level of p < .05.

To investigate how change or persistence from 4.5 to 8 years in behavioral difficulties total score is associated with cognitive performance at 8 years, a multivariate logistic regression analysis was conducted for each cognitive outcome (below average cognitive abilities vs. typical) while controlling for the aforementioned covariates.

Multicollinearity was assessed by calculating the generalised variance inflation factors (GVIF). Variables with VIF > 4, indicate a potential concern for multicollinearity [51].

Posthoc multiple comparisons between each level of the behavioral persistence/change variable were calculated using general linear hypothesis tests with a Tukey correction applied for multiple comparisons [52]. We also interpreted the effect sizes of significant odds ratios based on Cohen’s *d* effect sizes [53] and Chinn’s [54] method to convert odds ratios to effect size. Accordingly, effect size magnitudes as odds ratios are as follows: 1.47 = small effect; 2.47 = medium effect; and 4.25 = large effect.

## Results

*Descriptive statistics.* When compared to the *Growing Up in New Zealand* baseline sample (n = 6853), cases with missing data for the current analysis were less likely to be European, more likely to live in a rural, highly deprived area and to have mothers of lower education while the pregnancy was less likely to be planned (p < .001). No differences were found with respect to children’s sex . The distribution of children within the SDQ 4 band categorisation at 4.5 and 8 years are presented in Table 1. The distribution of the total difficulties scores dichotomized into average/slightly raised vs. high/very high at 4.5 and 8 years can be found in Table 2. Table 3 presents the frequency distribution of behavioral difficulties (close to average/slightly raised and high/very high) and control variables across each cognitive measure (below average vs. typical) at age 8.

**Table 1:** Distribution of children within the SDQ 4 band categorisation at 8 years

		4 band categorisation											
Behavioral difficulties		Close to average			Slightly raised			High			Very high		
		n	%		n	%		n	%		n	%	
Total difficulties 8 years		3423	87.7		235	6.0		143	3.7		103	2.6	
Total difficulties 4.5 years		3183	81.5		401	10.3		187	4.8		133	3.4	

**Table 2:** SDQ Total difficulties dichotomized at 8 years and 4.5 years

		Total cohort						
Behavioral difficulties		Close to average/ slightly raised			High/ Very high			
		n	%		n	%		
Total difficulties 8 years		3658	93.7		246	6.3		
Total difficulties 4.5 years		3584	91.8		320	8.2		

**Table 3:** Frequency distribution of behavioral difficulties and control variables across cognitive measure at 8 years

	Cognitive Outcome															
	Vocabulary		Inhibitory control			Working Memory		Cognitive Flexibility		Processing Speed			Episodic Memory		Reading	
Variable	Average and above	1 SD below average	Average and above	1 SD below average	Average and above		1 SD below average	Average and above	1 SD below average	Average and above	1 SD below average	Average and above		1 SD below average	Average and above	1 SD below average
Total difficulties																
Average/slightly raised	3238 (94.5)	420 (88.2)	3206 (94.6)	452 (87.9)	3246 (94.6)		412 (87.3)	3272 (94.1)	386 (90.2)	3133 (94.2)	525 (90.7)	3041 (94.5)		617 (89.9)	3200 (94.6)	458 (87.9)
High/very high	190 (5.5)	56 (11.8)	184 (5.4)	62 (12.1)	186 (5.4)		60 (12.7)	204 (5.9)	42 (9.8)	192 (5.8)	54 (9.3)	176 (5.5)		70 (10.2)	183 (5.4)	63 (12.1)
Emotional symptoms																
Average/slightly raised	3172 (92.5)	428 (89.9)	3144 (92.7)	456 (88.7)	3182 (92.7)		418 (88.6)	3209 (92.3)	391 (91.4)	3074 (92.5)	526 (90.8)	2976 (92.5)		624 (90.8)	3133 (92.6)	467 (89.6)
High/very high	256 (7.5)	48 (10.1)	246 (7.3)	58 (11.3)	250 (7.3)		54 (11.4)	267 (7.7)	37 (8.6)	251 (7.5)	53 (9.2)	241 (7.5)		63 (9.2)	250 (7.4)	54 (10.4)
Peer problems																
Average/slightly raised	3086 (90.0)	376 (79.0)	3026 (89.3)	436 (84.8)	3080 (89.7)		382 (80.9)	3108 (89.4)	354 (82.7)	2968 (89.3)	494 (85.3)	2880 (89.5)		582 (84.7)	3026 (89.4)	436 (83.7)
High/very high	342 (10.0)	100 (21.0)	364 (10.7)	78 (15.2)	352 (10.3)		90 (19.1)	368 (10.6)	74 (17.3)	357 (10.7)	85 (14.7)	337 (10.5)		105 (15.3)	357 (10.6)	85 (16.3)
Hyperactivity																
Average/slightly raised	3271 (95.4)	448 (94.1)	3228 (95.2)	491 (95.5)	3287 (95.8)		432 (91.5)	3330 (95.8)	389 (90.9)	3172 (95.4)	547 (94.5)	3079 (95.7)		640 (93.2)	3246 (96.0)	473 (90.8)
High/very high	157 (4.6)	28 (5.9)	162 (4.8)	23 (4.5)	145 (4.2)		40 (8.5)	146 (4.2)	39 (9.1)	153 (4.6)	32 (5.5)	138 (4.3)		47 (6.8)	137 (4.0)	48 (9.2)
Conduct problems																
Average/slightly raised	3168 (92.4)	409 (85.9)	3129 (92.3)	448 (87.2)	3169 (92.3)		408 (86.4)	3194 (91.9)	383 (89.5)	3057 (91.9)	520 (89.8)	2967 (92.2)		610 (88.8)	3124 (92.3)	453 (86.9)
High/very high	260 (7.6)	67 (14.1)	261 (7.7)	66 (12.8)	266 (7.7)		64 (13.6)	282 (8.1)	45 (10.5)	268 (8.1)	59 (10.2)	250 (7.8)		77 (11.2)	259 (7.7)	68 (13.1)
Ethnicity																
European	1594 (46.5)	101 (21.2)	1475 (43.5)	220 (42.8)	1523 (44.4)		172 (36.4)	1533 (44.1)	162 (37.9)	1454 (43.7)	241 (41.6)	1391 (43.2)		304 (44.3)	1475 (43.6)	220 (42.2)
Māori	739 (21.6)	162 (34.0)	769 (22.7)	132 (25.7)	736 (22.2)		138 (29.2)	778 (22.4)	123 (28.7)	745 (22.4)	156 (29.6)	730 (22.7)		171 (24.9)	721 (21.3)	180 (34.5)
Pacific Peoples	267 (7.8)	99 (20.8)	317 (9.4)	49 (9.5)	308 (9.0)		58 (12.3)	317 (9.1)	49 (11.4)	308 (9.3)	58 (10.0)	293 (9.1)		73 (10.6)	317 (9.4)	49 (9.4)
Asian	418 (12.2)	86 (18.1)	440 (13.0)	64 (12.5)	428 (12.5)		76 (16.1)	443 (12.7)	61 (14.3)	442 (13.3)	62 (10.7)	430 (13.4)		74 (10.8)	469 (13.9)	35 (6.7)
Other incl. MELAA	410 (11.9)	28 (5.9)	389 (11.5)	49 (11.4)	410 (12)		28 (6.0)	405 (11.6)	33 (7.7)	376 (11.3)	62 (10.7)	373 (11.5)		65 (9.5)	401 (11.8)	37 (7.2)
Mother’s education																
No secondary school	117 (3.5)	41 (8.8)	127 (3.8)	31 (6.2)	127 (3.8)		31 (6.8)	128 (3.8)	30 (7.2)	124 (3.8)	34 (6.0)	112 (3.6)		46 (6.9)	110 (3.3)	48 (9.5)
Secondary school/ Diploma/ Trade certificate	1588 (47.3)	298 (64.2)	1619 (48.7)	267 (53.6)	1609 (47.8)		277 (60.3)	1655 (48.6)	231 (55.3)	1579 (48.5)	307 (54.0)	1541 (48.9)		345 (51.5)	1595 (48.1)	291 (57.5)
Bachelor degree and higher	1653 (49.2)	125 (26.9)	1578 (47.5)	200 (40.2)	1627 (48.4)		151 (32.9)	1621 (47.6)	157 (37.6)	1551 (47.7)	227 (40.0)	1499 (47.6)		279 (41.6)	1611 (48.6)	167 (33.0)
Deprivation																
Low	1310 (38.8)	102 (21.7)	1252 (37.4)	160 (32.1)	1275 (37.7)		137 (29.4)	1262 (36.8)	150 (35.7)	1230 (37.6)	182 (31.8)	1178 (37.1)		234 (34.8)	1302 (39.1)	110 (21.4)
Medium	1352 (40.0)	155 (32.9)	1317 (39.3)	190 (38.2)	1337 (39.5)		170 (36.5)	1369 (39.9)	138 (32.9)	1258 (38.4)	249 (43.5)	1268 (39.9)		239 (35.6)	1290 (38.7)	217 (42.3)
High	714 (21.1)	214 (45.4)	780 (23.3)	148 (29.7)	769 (22.7)		159 (34.1)	796 (23.3)	132 (31.4)	787 (24.0)	141 (24.7)	729 (23.0)		199 (29.6)	742 (22.3)	186 (36.3)
Rurality																
Urban	2943 (87.2)	426 (90.4)	2926 (87.4)	443 (89.0)	2955 (87.4)		414 (88.8)	2993 (87.3)	376 (89.5)	2876 (87.8)	493 (86.2)	2783 (87.7)		586 (87.2)	2934 (88.0)	435 (84.8)
Rural	433 (12.8)	45 (9.6)	423 (12.5)	55 (11.0)	426 (12.6)		52 (11.2)	434 (12.4)	44 (10.5)	399 (12.2)	79 (13.8)	392 (12.3)		86 (12.8)	400 (12.0)	78 (15.2)
Mother’s age at birth																
<20 years	88 (2.6)	30 (6.4)	104 (3.1)	14 (2.8)	97 (2.9)		21 (4.5)	100 (2.9)	18 (4.3)	105 (3.2)	13 (2.3)	95 (3.0)		23 (3.4)	98 (3.0)	20 (3.9)
…20–29 years	1086 (32.3)	217 (42.6)	1109 (33.3)	187 (37.5)	1120 (33.3)		176 (38.1)	1135 (33.3)	161 (38.3)	1088 (33.4)	208 (36.6)	1063 (33.7)		233 (34.6)	1079 (32.5)	217 (42.6)
>29 years	2187 (65.1)	228 (48.7)	2117 (63.6)	298 (59.7)	2150 (63.9)		265 (57.4)	2174 (63.8)	241 (57.4)	2067 (63.4)	348 (61.2)	1998 (63.3)		417 (62.0)	2143 (64.5)	272 (53.4)
Pregnancy planned																
Yes	2336 (69.8)	253 (54.5)	2277 (68.7)	312 (63.0)	2288 (68.2)		301 (65.9)	2331 (68.6)	258 (62.3)	2203 (67.9)	386 (68.3)	2163 (68.8)		426 (64.0)	2301 (69.5)	288 (57.5)
No	1011 (30.2)	211 (45.5)	1039 (31.3)	183 (37.0)	1066 (31.8)		156 (34.1)	1066 (31.4)	156 (37.7)	1043 (32.1)	179 (31.7)	982 (31.2)		240 (36.0)	1009 (30.5)	213 (42.5)
Child’s sex																
Boy	1679 (49.0)	284 (59.7)	1710 (50.5)	253 (49.2)	1723 (50.2)		240 (50.8)	1709 (49.2)	254 (59.3)	1639 (49.3)	324 (56.0)	1603 (49.9)		360 (52.4)	1675 (49.5)	288 (55.4)
Girl	1747 (51.0)	192 (40.3)	1678 (49.5)	261 (50.8)	1707 (49.8)		232 (49.2)	1765 (50.8)	174 (40.7)	1684 (50.7)	255 (44.0)	1612 (50.1)		327 (47.6)	1707 (50.5)	232 (44.63)

Over time, 3417 (87.5%) of children had no difficulties at any time point, 241 (6.2%) children improved, 167 (4.3%) had a later onset of behavioral difficulties at 8 years and 79 (2.0%) showed persistent difficulties at both time points.

*Associations Between Persistence and Change in Behavioral Difficulties from 4.5 to 8 years and cognitive outcomes at 8 years.* The results of the multivariate logistic regression analyses can be found in Table 4. Compared to children with no difficulties, children with a later onset of behavioral difficulties showed an increased likelihood of below average vocabulary (OR = 1.73, p < .05), inhibitory control/attention (OR = 2.36, p < 001), working memory (OR = 2.14, p < .001), processing speed (OR = 1.76, p < .05), episodic memory (OR = 1.79, p < .01) and reading (OR = 1.75, p < .05). Furthermore, compared to children with no difficulties, children who had persistent difficulties at 4.5 and 8 years had increased odds of below average inhibitory control/attention (OR = 2.44, p < .01), working memory (OR = 2.29, p < .05), episodic memory (OR = 1.91, p < .05) and reading (OR = 2.12, p < 05) at 8 years. Additionally, children who had a later onset of behavioral difficulties showed increased odds of below average working memory compared to children who had improved (OR = 2.05, p < 05). While the effects were generally small in magnitude, the effects were strongest for the association between persistent behavioral difficulties and cognitive outcomes. Multicollinearity was not of concern (GVIFs ≤ 1.41).

**Table 4:** Multivariate logistic regression evaluating the association between persistence and change in behavioral difficulties and cognitive outcomes at 8 years (post-hoc multiple comparison)

Cognitive Outcome (1SD below mean vs average and above)																									
Behavioral difficulties	Vocabulary			Inhibitory control			Working Memory			Cognitive Flexibility				Processing Speed					Episodic Memory			Reading			
	B (SE)	OR (99% CI)	z value	B (SE)	OR (99% CI)	z value	B (SE)	OR (99% CI)	z value		B (SE)	OR (99% CI)	z value		B (SE)	OR (99% CI)		z value	B (SE)	OR (99% CI)	z value	B (SE)	OR (99% CI)	z value	
Improved vs no difficulties	0.35 (0.18)	1.42 0.89–2.28)	1.93	0.33 (0.20)	1.39 (0.83–2.30)	1.65	0.04 (0.21)	1.04 (0.61–1.79)	0.21		0.16 (0.21)	1.17 (0.68–2.01)	0.75		0.30 (0.19)	1.35 (0.83–2.19)		1.61	0.054 (0.18)	1.05 (0.65–1.68)	0.25	0.37 (0.20)	1.45 (0.87–2.41)	1.89	
Later onset vs no difficulties	**0.55 (0.21)**	**1.73** **(1.00–3.00)**	**2.56** ^*^	**0.86** **(0.19)**	**2.36** **(1.44–3.88)**	**4.46** ^***^	**0.76** **(0.20)**	**2.14** **(1.28–3.60)**	**3.80** ^***^		0.46 (0.22)	1.58 (0.89–2.78)	2.07		**0.56** **(0.19)**	**1.76** **(1.07–2.90)**		**2.91** ^*^	**0.58** **(0.18)**	**1.79** **(1.11–2.87)**	**3.16** ^**^	**0.56** **(0.20)**	**1.75** **(1.03–2.97)**	**2.73** ^*^	
Persistent vs no difficulties	0.52 (0.28)	1.69 (0.81–3.51)	1.85	**0.89** **(0.28)**	**2.44** **(1.20–4.96)**	**3.24** ^**^	**0.83** **(0.28)**	**2.29** **(1.12–4.69)**	**3.00** ^*^		0.22 (0.33)	1.25 (0.54–2.91)	0.68		0.17 (0.31)	1.19 (0.54–2.63)		0.56	**0.65** **(0.26)**	**1.91** **(0.97–3.74)**	**2.45** ^*^	**0.75** **(0.28)**	**2.12** **(1.04–4.35)**	**2.71** ^*^	
Later onset vs improved	0.20 (0.27)	1.22 (0.61–2.05)	0.73	0.53 (0.26)	1.70 (0.87–3.35)	2.03	**0.72** **(0.28)**	**2.05** **(1.00-4.20)**	2.60*		0.30 (0.29)	1.35 (0.64–2.85)	1.02		0.26 (0.26)	1.30 (0.67–2.53)		1.01	0.53 (0.24)	1.71 (0.90–3.25)	2.14	0.19 (0.27)	1.21 (0.60–2.42)	0.69	
Persistent vs improved	0.17 (0.32)	1.19 (0.52–2.73)	0.53	0.56 (0.32)	1.76 (0.76–4.05)	1.75	0.79 (0.33)	2.20 (0.93–5.17)	2.37		0.06 (0.38)	1.07 (0.41–2.80)	0.17		-0.13 (0.35)	0.88 (0.36–2.16)		-0.37	0.60 (0.31)	1.82 (0.83–4.02)	1.96	0.38 (0.33)	1.46 (0.63–3.38)	1.17	
Persistent vs later onset	-0.02 (0.34)	0.98 (0.40–2.37)	-0.07	0.03 (0.32)	1.03 (0.45–2.38)	0.10	0.07 (0.33)	1.07 (0.46–2.50)	0.20		-0.23 (0.38)	0.79 (0.29–2.13)	-0.61		-0.39 (0.36)	0.68 (0.27–1.69)		-1.10	0.06 (0.31)	1.07 (0.48–2.37)	0.21	0.19 (0.33)	1.21 (0.51–2.87)	0.58	

Note.^*^p < .05; ^**^p < .01; ^***^p < .001. Behavioral difficulties: High/very high compared to average/slightly raised; Low/very low compared to average/slightly lowered. All analyses controlled for mother’s ethnicity, mother’s education, mother’s age when pregnant, area-level deprivation, rurality, child’s age, child’s gender, planned pregnancy, time of testing. OR: 1.47 = small effect; 2.47 = medium effect; 4.25 = large effect

## Discussion

In the current study, we investigated the association between persistence and change in behavioral difficulties and cognitive performance in 8-year-old children, enrolled in the *Growing Up in New Zealand* study. Our findings are novel and important as we looked at behavior stability over time when transitioning from preschool age (4.5 years) to school age (8 years) and cognitive outcomes were assessed using a comprehensive standardized cognitive battery including measures of language, executive functioning, episodic memory, working memory and processing speed.

Compared to children with no difficulties, children who had persistent difficulties (behavioral difficulties both at 4.5 and 8 years) or who had a later onset (showing behavioral difficulties at 8 years only) performed more poorly across a range of cognitive outcomes at 8 years. Specifically, children who had a later onset of behavioral difficulties compared to children with no difficulties were more likely to perform below average in tests of inhibitory control/attention, working memory, processing speed, episodic memory, vocabulary and reading. Children who had persistent behavioral difficulties were at higher risk for below average cognitive abilities in the areas of inhibitory control/attention, working memory, reading and episodic memory. Additionally, children who had a later onset of behavioral difficulties had greater odds of below average working memory performance compared to children who had improved. The effect of these associations appeared to be strongest for inhibitory control/attention, followed by working memory which also showed the most associations with behavioural difficulties. No significant associations between behavioral difficulties and cognitive outcomes were found for cognitive flexibility. There was also no significant difference in children whose behavioral difficulties improved compared to children with no difficulties.

Our study is an extension of an earlier longitudinal study with the same cohort studied in early childhood. Consistent with our findings, the earlier work demonstrated that only those children with later onset or persistent behavioral difficulties had an increased likelihood of showing preschool cognitive delay (i.e. executive control, receptive language and early literacy; [20]). Here, when transitioning from preschool to age 8, we observed that later onset and persistent behavioral difficulties were associated with the likelihood of underperformance in a broader range of cognitive abilities. No association was found for children whose behavioral problems improved from age 4.5 to age 8 years.

Interestingly, we found more statistically significant associations between cognitive outcomes and later onset of behavioral difficulties than between cognitive outcomes and persistent behavioral difficulties. One explanation might be that the incidence of later behavioral difficulties may have been experienced as unsettling and thus affecting performance in several cognitive areas. For instance, newly occurring behavioral problems might have been associated with major life events or disruptions in the children’s lives which may have affected a broader range of cognitive abilities directly or indirectly [55, 56]. Likewise, it is possible that current difficulties in academic success might have impacted on current behavioral patterns as this relationship is likely to be bidirectional. In this regard, it appears also plausible that the concurrent nature of the behavioral problems is more relevant as it has a more direct impact on the NIH Toolbox CB performance. Another possibility is that the group of children with later-onset of behavioral difficulties may be acting out because their cognitive difficulties, along with school becoming more demanding during middle childhood, manifesting behavioral problems in order to compensate for cognitive obstacles and poorer grades [57].

At the same time, we found that within the same cognitive outcome effect sizes were slightly greater for children who had persistent behavioral difficulties compared with children who showed later onset of difficulties. An earlier study using the *Growing Up in New Zealand* data investigated the persistence and change in clinically relevant behavioral problems during early childhood and observed that those whose difficulties persist are also more likely to experience risk factors for vulnerability [26]. This is in line with a secondary analysis of data from the *UK Millennium Cohort Study* and *the Longitudinal Study of Australian Children* with 2- to 3-year-olds, which showed that higher rates of behavioral difficulties among children with developmental delay may be partially due to a greater likelihood of exposure to adverse socio-economic conditions [58]. Hence, persistent behavioral difficulties after transition to school are likely to be accompanied by additional challenges in the children’s lives. This supports D’Souza and colleague’s [20] assumption that ongoing behavioral problems may have a greater impact on children’s cognitive performance.

It should be noted that we are not using clinical markers of cognitive impairment. We have simply identified the lowest end of the normal distribution of cognitive outcomes of the NIH Toolbox CB. This dichotomisation was chosen rather than the continuous cognitive outcomes as we were interested how behavioral difficulties relate to the likelihood of poorer cognitive functioning as in contrast to cognitive performance per se. The classification of neuropsychological impairment is dependent on the normative comparison applied and there is no normative data available for the Aotearoa New Zealand population [31]. In a study to develop demographically corrected normative standards for the English Version of the NIH Toolbox CB within a U.S. sample, cut-points were calculated one standard deviation below the mean as an operational definition of “impairment” across the fully corrected composites to increase clinical interpretation in a sample of children (3–17 years) [59]. Likewise, we used the one standard deviation below the mean threshold to indicate poorer cognitive functioning in our sample.

Our results provide a deeper insight into the association between persistence and change in behavioral difficulties when transitioning from preschool into school with specific cognitive abilities at age 8. Specifically, we found that that persistent and later onset of behavioral difficulties was associated with a range of cognitive abilities. Our results, alongside the recent findings of D’Souza and colleagues [20], extend the existing literature by demonstrating that both persistent and later onset of behavioral problems are associated with below average cognitive performance in contrast to children who showed no or improved difficulties. However, we cannot account for causation as the relationship between cognitive difficulties and behavioral problems is likely to be bidirectional in nature [4, 15, 16] and may be mediated by other underlying factors such as genetic or environmental factors which affect the manifestation of both cognitive and behavioral difficulties [60–62].

Our findings have practical implications, as the SDQ is used in the New Zealand Health Survey and the B4 School Check and has been widely administered to screen for psychopathology in early childhood both internationally and in the Aotearoa New Zealand population [63, 64]. There has been increasing awareness regarding the social-emotional aspects of learning, indicating that young people must be socially and emotionally ready to learn in order to be able to benefit from the educational curriculum [65, 66]. Our study demonstrates that children with persistent and later onset behavioral difficulties are more likely to perform more poorly in several cognitive key outcomes which may potentially impact their future academic success. Given, we cannot account for any causal direction, there is a likely co-occurrence of challenges, with children who are struggling with the educational course also having to face behavioral difficulties. This highlights the importance of identifying and addressing behavioral problems alongside cognitive and educational difficulties to provide adequate support and resources for those requiring additional assistance [66].

Our study has several limitations. First, while one could infer that there may be some effect on behavioral difficulties when transitioning from pre-school to formal schooling, we did not directly measure this but captured this as a result of to the timing of measurement at preschool age and age 8 years. Consequently, many other factors could also have influenced the cognitive outcomes. In this regard, a methodological limitation is the lack of a baseline assessment of cognitive abilities due to the retrospective nature of this study. Thus, changes or stability in cognitive abilities over time as well as other factors not accounted for in this study such as the family environment and parenting factors may also partially accounted for associations between the children’s cognitive and behavioral outcomes [67, 68]. As the Growing Up in New Zealand study is an observational population-based study, some children may have naturally been in treatment for behavioral or cognitive problems which may have influenced the findings over time. Third, literature generally acknowledges that cognitive tests may be culturally biased with respect to their content and administration procedure [69, 70]. Fluid constructs, like attention/inhibitory control, might be less culturally biased than language tests but performance in these may also be dependent on the children’s ability to understand the instructions. In this regard, a proportion of the children in our sample are bilingual, with some having another language as English as their primary language which might have affected the cognitive test results. Additionally, the NIH Toolbox CB may not have been feasible for all children or suitable for those with special needs or requiring assistance. There may be some bias due to method effects, i.e. as the cognitive assessment was administered at the end of a longer interview after school, this may have caused an additional challenge for those with behavioral difficulties, especially for children with high hyperactivity. Furthermore, there may be bias when assessing behavior solely by mother-report as opposed to including father- and teacher-report or direct observation. [71] As cases with missing data showed a different socio-demographic distribution compared to the baseline sample, the overall generalizability is limited.

In conclusion, this study accounts for the longitudinal perspective of behavioral problems during the important stage of transitioning from preschool to school age and how persistence and change are linked to different cognitive areas at age 8 assessed with a comprehensive standardized cognitive battery. We found that children who showed persistent or later onset of behavioral difficulties were more likely to show below average performance across language, executive functioning, episodic memory, working memory and processing speed compared with children who had no difficulties across time. As cognitive performance is closely related to academic achievement, our study supports the importance of identifying and addressing needs in both cognitive skills and behavioral aspects when planning interventions and educational programmes in early and middle childhood.

### Summary

The current study investigated the association between persistence and change in behavioral difficulties during early to middle childhood and a range of cognitive outcomes at age 8. Our sample comprised 3904 8-year-old children enrolled in the longitudinal *GrowingUp in New Zealand *study. The NIH Toolbox CB was used to assess cognitive outcomes at 8 years including vocabulary, language, inhibitory control/attention, processing speed, cognitive flexibility, working memory and episodic memory. The parent administered SDQ was used to assess behavioral difficulties at 4.5 and 8 years. Multivariate logistic regression analyses were conducted with cognitive measures as outcomes and persistence/change in the SDQ Total difficulties score as predictors while controlling for a range of sociodemographic confounders. Our findings show that children with persistent or later onset of behavioral difficulties were at higher risk for poorer vocabulary, reading, inhibitory control/attention, episodic memory, working memory and processing speed at age 8 compared to children with no difficulties or improved difficulties. Even though we cannot account for causation and other factors that might have influenced cognitive performance, there is a likely co-occurrence of behavioral and cognitive challenges. Thus, our study highlights the importance of identifying and addressing both behavioral problems as well as cognitive and educational difficulties to provide adequate support and resources when planning educational programmes and interventions in early and middle childhood.

## References

[1] Rutter M, Rutter M, Taylor E (2002). Development and psychopathology. Child and adolescent psychiatry.

[2] Beitchman JH, Wilson B, Johnson CJ, Atkinson L, Young A, Adlaf E (2001). Fourteen-year follow-up of speech/language-impaired and control children: Psychiatric outcome. J Am Acad Child Adolesc Psychiatry.

[3] Benasich AA, Curtiss S, Tallal P (1993). Language, learning, and behavioral disturbances in childhood: a longitudinal perspective. J Am Acad Child Adolesc Psychiatry.

[4] Helland WA, Lundervold AJ, Heimann M, Posserud M-B (2014). Stable associations between behavioral problems and language impairments across childhood–The importance of pragmatic language problems. Res Dev Disabil.

[5] Gremillion ML, Martel MM (2014). Merely misunderstood? Receptive, expressive, and pragmatic language in young children with disruptive behavior disorders. J Clin Child Adolesc Psychol.

[6] Sim F, O’Dowd J, Thompson L, Law J, Macmillan S, Affleck M (2013). Language and social/emotional problems identified at a universal developmental assessment at 30 months. BMC Pediatr.

[7] Mackie L, Law J (2010). Pragmatic language and the child with emotional/behavioural difficulties (EBD): a pilot study exploring the interaction between behaviour and communication disability. Int J Lang communication disorders.

[8] Achenbach TM, Rescorla L (2001) Manual for the ASEBA school-age forms & profiles: An integrated system of multi-informant assessment Aseba., Burlington, VT

[9] Raaijmakers MA, Smidts DP, Sergeant JA, Maassen GH, Posthumus JA, Van Engeland H (2008). Executive functions in preschool children with aggressive behavior: Impairments in inhibitory control. J Abnorm Child Psychol.

[10] Jahromi LB, Stifter CA(2008) Individual differences in preschoolers’ self-regulation and theory of mind.Merrill-Palmer Quarterly:125–150

[11] Adams JW, Snowling MJ (2001). Executive function and reading impairments in children reported by their teachers as ‘hyperactive’. Br J Dev Psychol.

[12] Adams JW, Snowling MJ, Hennessy SM, Kind P (1999). Problems of behaviour, reading and arithmetic: Assessments of comorbidity using the Strengths and Difficulties Questionnaire. Br J Educ Psychol.

[13] Fergusson DM, Horwood LJ, Lynskey MT (1993). The effects of conduct disorder and attention deficit in middle childhood on offending and scholastic ability at age 13. J Child Psychol.

[14] McGee R, Prior M, Williams S, Smart D, Sanson A (2002). The long-term significance of teacher‐rated hyperactivity and reading ability in childhood: Findings from two longitudinal studies. J Child Psychol Psychiatry.

[15] Gallagher TM (1999). Interrelationships among children’s language, behavior, and emotional problems. Top Lang disorders.

[16] Redmond SM, Rice ML (1998). The socioemotional behaviors of children with SLI: Social adaptation or social deviance?. J Speech Lang Hear Res.

[17] DeBaryshe BD, Patterson GR, Capaldi DM (1993). A performance model for academic achievement in early adolescent boys. Dev Psychol.

[18] Ketelaars MP, Cuperus J, Jansonius K, Verhoeven L (2010). Pragmatic language impairment and associated behavioural problems. Int J Lang Commun Disord.

[19] Williams S, McGee R (1994). Reading attainment and juvenile delinquency. J Child Psychol Psychiatry.

[20] D’Souza S, Underwood L, Peterson ER, Morton SM, Waldie KE(2020) The Association Between Persistence and Change in Early Childhood Behavioural Problems and Preschool Cognitive Outcomes.Child Psychiatry Human Development:1–11 10.1007/s10578-019-00953-x 31907733

[21] Zelazo PD, Anderson JE, Richler J, Wallner-Allen K, Beaumont JL, Weintraub S (2013). II. NIH Toolbox Cognition Battery (CB): Measuring executive function and attention. Monogr Soc Res Child Dev.

[22] Vandenbroucke L, Verschueren K, Baeyens D (2017). The development of executive functioning across the transition to first grade and its predictive value for academic achievement. Learn Instruction.

[23] Hartas D (2011). Children’s language and behavioural, social and emotional difficulties and prosocial behaviour during the toddler years and at school entry. Br J Special Educ.

[24] Rose SL, Rose SA, Feldman JF (1989). Stability of behavior problems in very young children. Dev Psychopathol.

[25] Keenan K, Wakschlag L (2000). More than the terrible twos: The nature and severity of behavior problems in clinic-referred preschool children. J Abnorm Child Psychol.

[26] D’Souza S, Underwood L, Peterson ER, Morton SM, Waldie KE (2019). Persistence and change in behavioural problems during early childhood. BMC Pediatr.

[27] Morton SM, Grant CC, Atatoa Carr PE, Robinson EM, Kinloch JM, Fleming CJ (2014). How do you recruit and retain a prebirth cohort? Lessons learnt from Growing Up in New Zealand. Eval Health Prof.

[28] Morton SM, Ramke J, Kinloch J, Grant CC, Atatoa Carr PE, Leeson H (2015). Growing Up in New Zealand cohort alignment with all New Zealand births. Aust N Z J Public Health.

[29] Gershon RC, Cella D, Fox NA, Havlik RJ, Hendrie HC, Wagster MV (2010). Assessment of neurological and behavioural function: the NIH Toolbox. Lancet Neurol.

[30] Weintraub S, Bauer PJ, Zelazo PD, Wallner-Allen K, Dikmen SS, Heaton RK (2013). I. NIH Toolbox Cognition Battery (CB): introduction and pediatric data. Monogr Soc Res Child Dev.

[31] Neumann D, Peterson ER, Underwood L, Morton SMB, Waldie KE(2021) Exploring the factor structure of the NIH Toolbox Cognition Battery in a large sample of 8-year old children in Aotearoa New Zealand.J Int Neuropsychol Soc:1–10 10.1017/S1355617720001265 33423713

[32] Goodman R (1997). The Strengths and Difficulties Questionnaire: a research note. J Child Psychol Psychiatry.

[33] D’Souza S, Waldie KE, Peterson ER, Underwood L, Morton SM (2017). Psychometric properties and normative data for the preschool strengths and difficulties questionnaire in two-year-old children. J Abnorm Child Psychol.

[34] D’Souza S, Waldie KE, Peterson ER, Underwood L, Morton SM (2019). The strengths and difficulties questionnaire: factor structure of the father-report and parent agreement in 2-year-old children. Assessment.

[35] Youth in Mind (2014) SDQ: information for researchers and professionals about the Strengths and Difficulties Questionnaires (Internet). https://www.sdqinfo.org/. Accessed 23 Jul 2020

[36] Ministry of Health (2018) Social, Emotional and Behavioural Difficulties in New Zealand Children: Technical Report. Wellington: Ministry of Health

[37] D’Souza S, Crawford CN, Buckley J, Underwood L, Peterson ER, Bird A (2019). Antenatal determinants of early childhood talking delay and behavioural difficulties. Infant Behav Dev.

[38] Ministry of Health (2015) B4 School Check (Internet). https://www.health.govt.nz/our-work/life-stages/child-health/b4-school-check. Retrieved: 10.07.2020

[39] D’Souza S, Waldie KE, Peterson ER, Underwood L, Morton SM (2019). Antenatal and postnatal determinants of behavioural difficulties in early childhood: Evidence from growing up in New Zealand. Child Psychiatry Human Development.

[40] Buckley J, Peterson ER, Underwood L, D’Souza S, Morton S, Waldie KEJL et al(2020) Socio-demographic and maternal health indicators of inhibitory control in preschool age children: evidence from Growing Up in New Zealand. 11(2):181–201

[41] Neumann D, Herbert SE, Peterson ER, Underwood L, Morton SM, Waldie KE (2019). A longitudinal study of antenatal and perinatal risk factors in early childhood cognition: Evidence from Growing Up in New Zealand. Early Hum Dev.

[42] Statistics New Zealand (2004) Report of the Review of the Measurement of Ethnicity Statistics New Zealand, Wellington

[43] Statistics New Zealand (2005) Statistical Standard for Ethnicity Statistics New Zealand, Wellington

[44] Atatoa Carr P, Langridge F, Neumann D, Paine S-J, Liang R, Taufa S et al(2022) ‘Seeing’our tamariki in longitudinal studies: exploring the complexity of ethnic identification trajectories within Growing Up in New Zealand.Journal of the Royal Society of New Zealand:1–17 10.1080/03036758.2022.2064518 PMC11485874 39439586

[45] Yao ES, Meissel K, Bullen P, Carr PA, Clark TC, Morton SM (2021). Classifying multiple ethnic identifications. Demographic Res.

[46] Yao ES, Meissel K, Bullen P, Clark TC, Carr PA, Tiatia-Seath J (2022). Demographic discrepancies between administrative-prioritisation and self-prioritisation of multiple ethnic identifications. Soc Sci Res.

[47] Willingham DT (2012). Ask the Cognitive Scientist: Why Does Family Wealth Affect Learning?. Am Educ.

[48] Bradley RH, Corwyn RF (2002). Socioeconomic status and child development. Annu Rev Psychol.

[49] Atkinson J, Salmond C, Crampton P (2014). NZDep2013 index of deprivation.

[50] Statistics New Zealand (2004) An urban/rural profile Statistics New Zealand, Wellington

[51] Glantz SA, Slinker BK, Neilands TB (1990). Primer of applied regression and analysis of variance McGraw-Hill.

[52] Bretz F, Westfall P, Hothorn T(2016)Multiple comparisons using R CRC Press, London

[53] Cohen J (2013). Statistical power analysis for the behavioral sciences.

[54] Chinn S (2000). A simple method for converting an odds ratio to effect size for use in meta-analysis. Stat Med.

[55] Berden GF, Althaus M, Verhulst FC, Psychiatry (1990). Major life events and changes in the behavioural functioning of children. J Child Psychol Psychiatry.

[56] Flouri E, Mavroveli S, Panourgia C (2013). The role of general cognitive ability in moderating the relation of adverse life events to emotional and behavioural problems. Br J Psychol.

[57] Waldie K, Spreen O (1993). The relationship between learning disabilities and persisting delinquency. J Learn Disabil.

[58] Emerson E, Einfeld S (2010). Emotional and behavioural difficulties in young children with and without developmental delay: a bi-national perspective. J Child Psychol Psychiatry.

[59] Casaletto KB, Umlauf A, Beaumont J, Gershon R, Slotkin J, Akshoomoff N (2015). Demographically corrected normative standards for the English version of the NIH Toolbox Cognition Battery. J Int Neuropsychol Soc.

[60] Savitz J, Solms M, Ramesar R, Genes (2006). The molecular genetics of cognition: dopamine, COMT and BDNF. Brain and Behavior.

[61] Van den Bergh BR, Mulder EJ, Mennes M, Glover V (2005). Antenatal maternal anxiety and stress and the neurobehavioural development of the fetus and child: links and possible mechanisms. A review. Neurosci Biobehav Rev.

[62] Caspi A, Moffitt TE (2018). All for one and one for all: Mental disorders in one dimension. Am J Psychiatry.

[63] Slykerman RF, Thompson J, Waldie KE, Murphy R, Wall C, Mitchell EA (2017). Antibiotics in the first year of life and subsequent neurocognitive outcomes. Acta Paediatr.

[64] Thompson JM, Sonuga-Barke EJ, Morgan AR, Cornforth CM, Turic D, Ferguson LR (2012). The catechol‐O‐methyltransferase (COMT) Val158Met polymorphism moderates the effect of antenatal stress on childhood behavioural problems: longitudinal evidence across multiple ages. Dev Med Child Neurol.

[65] Turkstra LS, Williams WH, Tonks J, Frampton I (2008). Measuring social cognition in adolescents: Implications for students with TBI returning to school. NeuroRehabilitation.

[66] Cortina MA, Fazel M (2015). The Art Room: An evaluation of a targeted school-based group intervention for students with emotional and behavioural difficulties. The Arts in Psychotherapy.

[67] Raviv T, Kessenich M, Morrison FJ (2004). A mediational model of the association between socioeconomic status and three-year-old language abilities: The role of parenting factors. Early Child Res Q.

[68] Schroeder VM, Kelley ML (2010). Family environment and parent-child relationships as related to executive functioning in children. Early child development care.

[69] Ogden JA, McFarlane-Nathan G (1997). Cultural bias in the neuropsychological assessment of young Māori men. NZ J Psychol.

[70] Haitana T, Pitama S, Rucklidge JJ (2010). Cultural biases in the peabody picture vocabulary test-III: testing tamariki in a New Zealand sample. NZ J Psychol.

[71] Mieloo CL, Bevaart F, Donker MC, van Oort FV, Raat H, Jansen W (2014). Validation of the SDQ in a multi-ethnic population of young children. Eur J Public Health.

[72] Raaijmakers MA, Smidts DP, Sergeant JA, Maassen GH, Posthumus JA, Van Engeland H, et al. (2008) Executive functions in preschool children with aggressive behavior: Impairments in inhibitory control. J Abnorm Child Psychol 36(7):1097 10.1007/s10802-008-9235-7 18437548

